# Internet Addiction: A Brief Summary of Research and Practice

**DOI:** 10.2174/157340012803520513

**Published:** 2012-11

**Authors:** Hilarie Cash, Cosette D Rae, Ann H Steel, Alexander Winkler

**Affiliations:** a reSTART Internet Addiction Recovery Program, Fall City, WA 98024; b University of Marburg, Department for Clinical Psychology and Psychotherapy, Gutenbergstraße 18, 35032 Marburg, Germany

**Keywords:** Addiction, Computer, Internet, reSTART, Treatment.

## Abstract

Problematic computer use is a growing social issue which is being debated worldwide. Internet Addiction Disorder (IAD) ruins lives by causing neurological complications, psychological disturbances, and social problems. Surveys in the United States and Europe have indicated alarming prevalence rates between 1.5 and 8.2% [1]. There are several reviews addressing the definition, classification, assessment, epidemiology, and co-morbidity of IAD [2-5], and some reviews [6-8] addressing the treatment of IAD. The aim of this paper is to give a preferably brief overview of research on IAD and theoretical considerations from a practical perspective based on years of daily work with clients suffering from Internet addiction. Furthermore, with this paper we intend to bring in practical experience in the debate about the eventual inclusion of IAD in the next version of the Diagnostic and Statistical Manual of Mental Disorders (DSM).

## INTRODUCTION

The idea that problematic computer use meets criteria for an addiction, and therefore should be included in the next iteration of the *Diagnostic and Statistical Manual of Mental Disorders (DSM)*, 4^th^ ed. Text Revision [9] was first proposed by Kimberly Young, PhD in her seminal 1996 paper [10]. Since that time IAD has been extensively studied and is indeed, currently under consideration for inclusion in the *DSM-V* [11]. Meanwhile, both China and South Korea have identified Internet addiction as a significant public health threat and both countries support education, research and treatment [12]. In the United States, despite a growing body of research, and treatment for the disorder available in out-patient and in-patient settings, there has been no formal governmental response to the issue of Internet addiction. While the debate goes on about whether or not the DSM-V should designate Internet addiction a mental disorder [12-14] people currently suffering from Internet addiction are seeking treatment. Because of our experience we support the development of uniform diagnostic criteria and the inclusion of IAD in the *DSM-V* [11] in order to advance public education, diagnosis and treatment of this important disorder.

## CLASSIFICATION

There is ongoing debate about how best to classify the behavior which is characterized by many hours spent in non-work technology-related computer/Internet/video game activities [15]. It is accompanied by changes in mood, preoccupation with the Internet and digital media, the inability to control the amount of time spent interfacing with digital technology, the need for more time or a new game to achieve a desired mood, withdrawal symptoms when not engaged, and a continuation of the behavior despite family conflict, a diminishing social life and adverse work or academic consequences [2, 16, 17]. Some researchers and mental health practitioners see excessive Internet use as a symptom of another disorder such as anxiety or depression rather than a separate entity [e.g. 18]. Internet addiction could be considered an Impulse control disorder (not otherwise specified). Yet there is a growing consensus that this constellation of symptoms is an addiction [e.g. 19]. The *American Society of Addiction Medicine* (ASAM) recently released a new definition of addiction as a chronic brain disorder, officially proposing for the first time that addiction is not limited to substance use [20]. All addictions, whether chemical or behavioral, share certain characteristics including salience, compulsive use (loss of control), mood modification and the alleviation of distress, tolerance and withdrawal, and the continuation despite negative consequences. 

## DIAGNOSTIC CRITERIA FOR IAD

The first serious proposal for diagnostic criteria was advanced in 1996 by Dr. Young, modifying the DSM-IV criteria for pathological gambling [10]. Since then variations in both name and criteria have been put forward to capture the problem, which is now most popularly known as Internet Addiction Disorder. Problematic Internet Use (PIU) [21], computer addiction, Internet dependence [22], compulsive Internet use, pathological Internet use [23], and many other labels can be found in the literature. Likewise a variety of often overlapping criteria have been proposed and studied, some of which have been validated. However, empirical studies provide an inconsistent set of criteria to define Internet addiction [24]. For an overview see Byun *et al*. [25].

Beard [2] recommends that the following five diagnostic criteria are required for a diagnosis of Internet addiction: (1) Is preoccupied with the Internet (thinks about previous online activity or anticipate next online session); (2) Needs to use the Internet with increased amounts of time in order to achieve satisfaction; (3) Has made unsuccessful efforts to control, cut back, or stop Internet use; (4) Is restless, moody, depressed, or irritable when attempting to cut down or stop Internet use; (5) Has stayed online longer than originally intended. Additionally, at least one of the following must be present: (6) Has jeopardized or risked the loss of a significant relationship, job, educational or career opportunity because of the Internet; (7) Has lied to family members, therapist, or others to conceal the extent of involvement with the Internet; (8) Uses the Internet as a way of escaping from problems or of relieving a dysphoric mood (e.g., feelings of helplessness, guilt, anxiety, depression) [2].

There has been also been a variety of assessment tools used in evaluation. Young’s Internet Addiction Test [16], the Problematic Internet Use Questionnaire (PIUQ) developed by Demetrovics, Szeredi, and Pozsa [26] and the Compulsive Internet Use Scale (CIUS) [27] are all examples of instruments to assess for this disorder. 

## PREVALENCE

The considerable variance of the prevalence rates reported for IAD (between 0.3% and 38%) [28] may be attributable to the fact that diagnostic criteria and assessment questionnaires used for diagnosis vary between countries and studies often use highly selective samples of online surveys [7]. In their review Weinstein and Lejoyeux [1] report that surveys in the United States and Europe have indicated prevalence rates varying between 1.5% and 8.2%. Other reports place the rates between 6% and 18.5% [29].

“Some obvious differences with respect to the methodologies, cultural factors, outcomes and assessment tools forming the basis for these prevalence rates notwithstanding, the rates we encountered were generally high and sometimes alarming.” [24]

## ETIOLOGY

There are different models available for the development and maintenance of IAD like the cognitive-behavioral model of problematic Internet use [21], the anonymity, convenience and escape (ACE) model [30], the access, affordability, anonymity (Triple-A) engine [31], a phases model of pathological Internet use by Grohol [32], and a comprehensive model of the development and maintenance of Internet addiction by Winkler & Dörsing [24], which takes into account socio-cultural factors (*e.g.*, demographic factors, access to and acceptance of the Internet), biological vulnerabilities (*e.g.*, genetic factors, abnormalities in neurochemical processes), psychological predispositions (*e.g.*, personality characteristics, negative affects), and specific attributes of the Internet to explain “excessive engagement in Internet activities” [24]. 

## NEUROBIOLOGICAL VULNERABILITIES

It is known that addictions activate a combination of sites in the brain associated with pleasure, known together as the “reward center” or “pleasure pathway” of the brain [33, 34]. When activated, dopamine release is increased, along with opiates and other neurochemicals. Over time, the associated receptors may be affected, producing tolerance or the need for increasing stimulation of the reward center to produce a “high” and the subsequent characteristic behavior patterns needed to avoid withdrawal. Internet use may also lead specifically to dopamine release in the nucleus accumbens [35, 36], one of the reward structures of the brain specifically involved in other addictions [20]. An example of the rewarding nature of digital technology use may be captured in the following statement by a 21 year-old male in treatment for IAD:

“I feel technology has brought so much joy into my life. No other activity relaxes me or stimulates me like technology. However, when depression hits, I tend to use technology as a way of retreating and isolating.”

## REINFORCEMENT/REWARD

What is so rewarding about Internet and video game use that it could become an addiction? The theory is that digital technology users experience multiple layers of reward when they use various computer applications. The Internet functions on a variable ratio reinforcement schedule (VRRS), as does gambling [29]. Whatever the application (general surfing, pornography, chat rooms, message boards, social networking sites, video games, email, texting, cloud applications and games, etc.), these activities support unpredictable and variable reward structures. The reward experienced is intensified when combined with mood enhancing/stimulating content. Examples of this would be pornography (sexual stimulation), video games (e.g. various social rewards, identification with a hero, immersive graphics), dating sites (romantic fantasy), online poker (financial) and special interest chat rooms or message boards (sense of belonging) [29, 37].

## BIOLOGICAL PREDISPOSITION

There is increasing evidence that there can be a genetic predisposition to addictive behaviors [38, 39]. The theory is that individuals with this predisposition do not have an adequate number of dopamine receptors or have an insufficient amount of serotonin/dopamine [2], thereby having difficulty experiencing normal levels of pleasure in activities that most people would find rewarding. To increase pleasure, these individuals are more likely to seek greater than average engagement in behaviors that stimulate an increase in dopamine, effectively giving them more reward but placing them at higher risk for addiction. 

## MENTAL HEALTH VULNERABILITIES

Many researchers and clinicians have noted that a variety of mental disorders co-occur with IAD. There is debate about which came first, the addiction or the co-occurring disorder [18, 40]. The study by Dong *et al*. [40] had at least the potential to clarify this question, reporting that higher scores for depression, anxiety, hostility, interpersonal sensitivity, and psychoticism were consequences of IAD. But due to the limitations of the study further research is necessary. 

## THE TREATMENT OF INTERNET ADDICTION

There is a general consensus that total abstinence from the Internet should not be the goal of the interventions and that instead, an abstinence from problematic applications and a controlled and balanced Internet usage should be achieved [6]. The following paragraphs illustrate the various treatment options for IAD that exist today. Unless studies examining the efficacy of the illustrated treatments are not available, findings on the efficacy of the presented treatments are also provided. Unfortunately, most of the treatment studies were of low methodological quality and used an intra-group design.

The general lack of treatment studies notwithstanding, there are treatment guidelines reported by clinicians working in the field of IAD. In her book “Internet Addiction: Symptoms, Evaluation, and Treatment”, Young [41] offers some treatment strategies which are already known from the cognitive-behavioral approach: (a) practice opposite time of Internet use (discover patient’s patterns of Internet use and disrupt these patterns by suggesting new schedules), (b) use external stoppers (real events or activities prompting the patient to log off), (c) set goals (with regard to the amount of time), (d) abstain from a particular application (that the client is unable to control), (e) use reminder cards (cues that remind the patient of the costs of IAD and benefits of breaking it), (f) develop a personal inventory (shows all the activities that the patient used to engage in or can’t find the time due to IAD), (g) enter a support group (compensates for a lack of social support), and (h) engage in family therapy (addresses relational problems in the family) [41]. Unfortunately, clinical evidence for the efficacy of these strategies is not mentioned.

### Non-psychological Approaches

Some authors examine pharmacological interventions for IAD, perhaps due to the fact that clinicians use psychopharmacology to treat IAD despite the lack of treatment studies addressing the efficacy of pharmacological treatments. In particular, selective serotonin-reuptake inhibitors (SSRIs) have been used because of the co-morbid psychiatric symptoms of IAD (e.g. depression and anxiety) for which SSRIs have been found to be effective [42-46]. Escitalopram (a SSRI) was used by Dell’Osso *et al*. [47] to treat 14 subjects with impulsive-compulsive Internet usage disorder. Internet usage decreased significantly from a mean of 36.8 hours/week to a baseline of 16.5 hours/week. In another study Han, Hwang, and Renshaw [48] used bupropion (a non-tricyclic antidepressant) and found a decrease of craving for Internet video game play, total game play time, and cue-induced brain activity in dorsolateral prefrontal cortex after a six week period of bupropion sustained release treatment. Methylphenidate (a psycho stimulant drug) was used by Han *et al*. [49] to treat 62 Internet video game-playing children diagnosed with attention-deficit hyperactivity disorder. After eight weeks of treatment, the YIAS-K scores and Internet usage times were significantly reduced and the authors cautiously suggest that methylphenidate might be evaluated as a potential treatment of IAD. According to a study by Shapira *et al*. [50], mood stabilizers might also improve the symptoms of IAD. In addition to these studies, there are some case reports of patients treated with escitalopram [45], citalopram (SSRI)- quetiapine (antipsychotic) combination [43] and naltrexone (an opioid receptor antagonist) [51].

A few authors mentioned that physical exercise could compensate the decrease of the dopamine level due to decreased online usage [52]. In addition, sports exercise prescriptions used in the course of cognitive behavioral group therapy may enhance the effect of the intervention for IAD [53].

### Psychological Approaches

Motivational interviewing (MI) is a client-centered yet directive method for enhancing intrinsic motivation to change by exploring and resolving client ambivalence [54]. It was developed to help individuals give up addictive behaviors and learn new behavioral skills, using techniques such as open-ended questions, reflective listening, affirmation, and summarization to help individuals express their concerns about change [55]. Unfortunately, there are currently no studies addressing the efficacy of MI in treating IAD, but MI seems to be moderately effective in the areas of alcohol, drug addiction, and diet/exercise problems [56].

Peukert *et al*. [7] suggest that interventions with family members or other relatives like “Community Reinforcement and Family Training” [57] could be useful in enhancing the motivation of an addict to cut back on Internet use, although the reviewers remark that control studies with relatives do not exist to date.

Reality therapy (RT) is supposed to encourage individuals to choose to improve their lives by committing to change their behavior. It includes sessions to show clients that addiction is a choice and to give them training in time management; it also introduces alternative activities to the problematic behavior [58]. According to Kim [58], RT is a core addiction recovery tool that offers a wide variety of uses as a treatment for addictive disorders such as drugs, sex, food, and works as well for the Internet. In his RT group counseling program treatment study, Kim [59] found that the treatment program effectively reduced addiction level and improved self-esteem of 25 Internet-addicted university students in Korea.

Twohig and Crosby [60] used an Acceptance & Commitment Therapy (ACT) protocol including several exercises adjusted to better fit the issues with which the sample struggles to treat six adult males suffering from problematic Internet pornography viewing. The treatment resulted in an 85% reduction in viewing at post-treatment with results being maintained at the three month follow-up (83% reduction in viewing pornography).

Widyanto and Griffith [8] report that most of the treatments employed so far had utilized a cognitive-behavioral approach. The case for using cognitive-behavioral therapy (CBT) is justified due to the good results in the treatment of other behavioral addictions/impulse-control disorders, such as pathological gambling, compulsive shopping, bulimia nervosa, and binge eating-disorders [61]. Wölfling [5] described a predominantly behavioral group treatment including identification of sustaining conditions, establishing of intrinsic motivation to reduce the amount of time being online, learning alternative behaviors, engagement in new social real-life contacts, psycho-education and exposure therapy, but unfortunately clinical evidence for the efficacy of these strategies is not mentioned. In her study, Young [62] used CBT to treat 114 clients suffering from IAD and found that participants were better able to manage their presenting problems post-treatment, showing improved motivation to stop abusing the Internet, improved ability to control their computer use, improved ability to function in offline relationships, improved ability to abstain from sexually explicit online material, improved ability to engage in offline activities, and improved ability to achieve sobriety from problematic applications. Cao, Su and Gao [63] investigated the effect of group CBT on 29 middle school students with IAD and found that IAD scores of the experimental group were lower than of the control group after treatment. The authors also reported improvement in psychological function. Thirty-eight adolescents with IAD were treated with CBT designed particularly for addicted adolescents by Li and Dai [64]. They found that CBT has good effects on the adolescents with IAD (CIAS scores in the therapy group were significant lower than that in the control group). In the experimental group the scores of depression, anxiety, compulsiveness, self-blame, illusion, and retreat were significantly decreased after treatment. Zhu, Jin, and Zhong [65] compared CBT and electro acupuncture (EA) plus CBT assigning forty-seven patients with IAD to one of the two groups respectively. The authors found that CBT alone or combined with EA can significantly reduce the score of IAD and anxiety on a self-rating scale and improve self-conscious health status in patients with IAD, but the effect obtained by the combined therapy was better. 

### Multimodal Treatments

A multimodal treatment approach is characterized by the implementation of several different types of treatment in some cases even from different disciplines such as pharmacology, psychotherapy and family counseling simultaneously or sequentially. Orzack and Orzack [66] mentioned that treatments for IAD need to be multidisciplinary including CBT, psychotropic medication, family therapy, and case managers, because of the complexity of these patients’ problems.

In their treatment study, Du, Jiang, and Vance [67] found that multimodal school-based group CBT (including parent training, teacher education, and group CBT) was effective for adolescents with IAD (n = 23), particularly in improving emotional state and regulation ability, behavioral and self-management style. The effect of another multimodal intervention consisting of solution-focused brief therapy (SFBT), family therapy, and CT was investigated among 52 adolescents with IAD in China. After three months of treatment, the scores on an IAD scale (IAD-DQ), the scores on the SCL-90, and the amount of time spent online decreased significantly [68]. Orzack *et al*. [69] used a psychoeducational program, which combines psychodynamic and cognitive-behavioral theoretical perspectives, using a combination of Readiness to Change (RtC), CBT and MI interventions to treat a group of 35 men involved in problematic Internet-enabled sexual behavior (IESB). In this group treatment, the quality of life increased and the level of depressive symptoms decreased after 16 (weekly) treatment sessions, but the level of problematic Internet use failed to decrease significantly [69]. Internet addiction related symptom scores significantly decreased after a group of 23 middle school students with IAD were treated with Behavioral Therapy (BT) or CT, detoxification treatment, psychosocial rehabilitation, personality modeling and parent training [70]. Therefore, the authors concluded that psychotherapy, in particular CT and BT were effective in treating middle school students with IAD. Shek, Tang, and Lo [71] described a multi-level counseling program designed for young people with IAD based on the responses of 59 clients. Findings of this study suggest this multi-level counseling program (including counseling, MI, family perspective, case work and group work) is promising to help young people with IAD. Internet addiction symptom scores significantly decreased, but the program failed to increase psychological well-being significantly. A six-week group counseling program (including CBT, social competence training, training of self-control strategies and training of communication skills) was shown to be effective on 24 Internet-addicted college students in China [72]. The authors reported that the adapted CIAS-R scores of the experimental group were significantly lower than those of the control group post-treatment.

### The reSTART Program

The authors of this article are currently, or have been, affiliated with the reSTART: Internet Addiction Recovery Program [73] in Fall City, Washington. The reSTART program is an inpatient Internet addiction recovery program which integrates technology detoxification (no technology for 45 to 90 days), drug and alcohol treatment, 12 step work, cognitive behavioral therapy (CBT), experiential adventure based therapy, Acceptance and Commitment therapy (ACT), brain enhancing interventions, animal assisted therapy, motivational interviewing (MI), mindfulness based relapse prevention (MBRP), Mindfulness based stress reduction (MBSR), interpersonal group psychotherapy, individual psychotherapy, individualized treatments for co-occurring disorders, psycho- educational groups (life visioning, addiction education, communication and assertiveness training, social skills, life skills, Life balance plan), aftercare treatments (monitoring of technology use, ongoing psychotherapy and group work), and continuing care (outpatient treatment) in an individualized, holistic approach. 

The first results from an ongoing OQ45.2 [74] study (a self-reported measurement of subjective discomfort, interpersonal relationships and social role performance assessed on a weekly basis) of the short-term impact on 19 adults who complete the 45+ days program showed an improved score after treatment. Seventy-four percent of participants showed significant clinical improvement, 21% of participants showed no reliable change, and 5% deteriorated. The results have to be regarded as preliminary due to the small study sample, the self-report measurement and the lack of a control group. Despite these limitations, there is evidence that the program is responsible for most of the improvements demonstrated.

## CONCLUSION

As can be seen from this brief review, the field of Internet addiction is advancing rapidly even without its official recognition as a separate and distinct behavioral addiction and with continuing disagreement over diagnostic criteria. The ongoing debate whether IAD should be classified as an (behavioral) addiction, an impulse-control disorder or even an obsessive compulsive disorder cannot be satisfactorily resolved in this paper. But the symptoms we observed in clinical practice show a great deal of overlap with the symptoms commonly associated with (behavioral) addictions. Also it remains unclear to this day whether the underlying mechanisms responsible for the addictive behavior are the same in different types of IAD (e.g., online sexual addiction, online gaming, and excessive surfing). From our practical perspective the different shapes of IAD fit in one category, due to various Internet specific commonalities (e.g., anonymity, riskless interaction), commonalities in the underlying behavior (e.g., avoidance, fear, pleasure, entertainment) and overlapping symptoms (e.g., the increased amount of time spent online, preoccupation and other signs of addiction). Nevertheless more research has to be done to substantiate our clinical impression.

Despite several methodological limitations, the strength of this work in comparison to other reviews in the international body of literature addressing the definition, classification, assessment, epidemiology, and co-morbidity of IAD [2-5], and to reviews [6-8] addressing the treatment of IAD, is that it connects theoretical considerations with the clinical practice of interdisciplinary mental health experts working for years in the field of Internet addiction. Furthermore, the current work gives a good overview of the current state of research in the field of internet addiction treatment. Despite the limitations stated above this work gives a brief overview of the current state of research on IAD from a practical perspective and can therefore be seen as an important and helpful paper for further research as well as for clinical practice in particular.
